# Transfer and Enzyme-Mediated Metabolism of Oxidized Phosphatidylcholine and Lysophosphatidylcholine between Low- and High-Density Lipoproteins

**DOI:** 10.3390/antiox9111045

**Published:** 2020-10-26

**Authors:** Naoko Sawada, Takashi Obama, Mirei Mizuno, Kiyoshi Fukuhara, Sanju Iwamoto, Toshihiro Aiuchi, Tomohiko Makiyama, Hiroyuki Itabe

**Affiliations:** 1 Division of Biological Chemistry, Department of Pharmaceutical Sciences, Showa University School of Pharmacy, 1-5-8 Hatanodai, Shinagawa-ku, Tokyo 142-8555, Japan; sasabe@pharm.showa-u.ac.jp (N.S.); obama@pharm.showa-u.ac.jp (T.O.); aiuchi@gmail.com (T.A.); t-maki@pharm.showa-u.ac.jp (T.M.); 2 Division of Medicinal Chemistry, Department of Pharmaceutical Sciences, Showa University School of Pharmacy, 1-5-8 Hatanodai, Shinagawa-ku, Tokyo 142-8555, Japan; mizuno@pharm.showa-u.ac.jp (M.M.); fukuhara@pharm.showa-u.ac.jp (K.F.); 3 Division of Physiology and Pathology, Department of Pharmacology, Toxicology and Therapeutics, Showa University School of Pharmacy, 1-5-8 Hatanodai, Shinagawa-ku, Tokyo 142-8555, Japan; iwasanju@med.showa-u.ac.jp

**Keywords:** oxidized phosphatidylcholine, lysophosphatidylcholine, HDL, oxLDL, Lp-PLA_2_, LCAT, LC-MS/MS

## Abstract

Oxidized low-density lipoprotein (oxLDL) and oxidized high-density lipoprotein (oxHDL), known as risk factors for cardiovascular disease, have been observed in plasma and atheromatous plaques. In a previous study, the content of oxidized phosphatidylcholine (oxPC) and lysophosphatidylcholine (lysoPC) species stayed constant in isolated in vivo oxLDL but increased in copper-induced oxLDL in vitro. In this study, we prepared synthetic deuterium-labeled 1-palmitoyl lysoPC and palmitoyl-glutaroyl PC (PGPC), a short chain-oxPC to elucidate the metabolic fate of oxPC and lysoPC in oxLDL in the presence of HDL. When LDL preloaded with d_13_-lysoPC was mixed with HDL, d_13_-lysoPC was recovered in both the LDL and HDL fractions equally. d_13_-LysoPC decreased by 50% after 4 h of incubation, while d_13_-PC increased in both fractions. Diacyl-PC production was abolished by an inhibitor of lecithin-cholesterol acyltransferase (LCAT). When d_13_-PGPC-preloaded LDL was incubated with HDL, d_13_-PGPC was transferred to HDL in a dose-dependent manner when both LCAT and lipoprotein-associated phospholipase A_2_ (Lp-PLA_2_) were inhibited. Lp-PLA_2_ in both HDL and LDL was responsible for the hydrolysis of d_13_-PGPC. These results suggest that short chain-oxPC and lysoPC can transfer between lipoproteins quickly and can be enzymatically converted from oxPC to lysoPC and from lysoPC to diacyl-PC in the presence of HDL.

## 1. Introduction

Lipoproteins modified by oxidation, including oxidized low-density lipoprotein (oxLDL) and oxidized high-density lipoprotein (oxHDL), are crucial for atherosclerosis development. The involvement of oxLDL in atherogenesis has been extensively investigated [1,2,3]. The presence of oxLDL in atheromatous plaques as well as in the circulation has been demonstrated immunologically, where an increase in circulating oxLDL levels was found to be associated with cardiovascular disease [4,5,6,7,8]. In addition, increased circulating oxHDL in patients with cardiovascular diseases and its accumulation in atherosclerotic lesions were also recently demonstrated [9,10].

The modification of oxLDL is heterogeneous, where polyunsaturated fatty acid (PUFA)-containing phospholipids are oxidized to form oxidized phospholipids and apolipoproteins are covalently modified with various lipid oxidation products, including malondialdehyde [11,12]. Various oxidized phosphatidylcholine (oxPC) species have been identified in oxLDL using mass spectrometry (MS) [2,13]. Lysophosphatidylcholine (lysoPC) is formed in oxLDL and may be responsible for some of the biological functions of oxLDL including the proliferation of macrophages and smooth muscle cells and the production of inflammatory cytokines [14,15].

Copper-induced oxLDL is used worldwide as an oxLDL model because of its simple procedure and reproducibility. Molecular species of oxPC produced in oxLDL in vitro were investigated using LC-MS/MS [16,17]. As a result, PC species containing PUFAs were found to decrease as the oxidation reactions proceeded. When LDL was oxidized with copper sulfate for 3 h, various oxPC species were generated, and the amount of lysoPC reached as much as 30% of all PC. The accumulation of lysoPC was abolished by pretreatment of LDL with an inhibitor of lipoprotein-associated phospholipase A_2_ (Lp-PLA_2_). Lp-PLA_2_ has a unique substrate specificity that preferentially hydrolyzes short and/or hydrophilic acyl chains at the *sn*-2 position [18]. Therefore, lysoPC is generated during the oxidation of LDL by the hydrolysis of short-chain oxPC molecules by Lp-PLA_2_.

In a recent study, we separated in vivo oxLDL from human plasma and observed the novel features of oxLDL [10]. As a result, at least two types of in vivo oxLDL were separated by anion-exchange column chromatography, one of which was negatively charged particles and increased by three-fold in the plasma obtained from patients with acute myocardial infarction compared to healthy plasma. This in vivo oxLDL had unique characteristics; first, its apoB-100 was modified with oxidized products, including oxPC; second, it coeluted with oxHDL indicating that oxLDL could interact with oxHDL particles in this fraction; third, the binding of oxLDL and oxHDL was not covalent but electrostatic. These observations suggest that HDL may be involved in the generation of oxLDL in vivo.

Interestingly, the phospholipid profiles of in vivo oxLDL showed little accumulation of oxPC and lysoPC, which did not differ from that of normal LDL. To elucidate the molecular mechanisms responsible for the generation of oxLDL in vivo, we examined the behavior of phospholipids produced during LDL oxidation.

In this study, we prepared two stable isotope-labeled PC species, namely 1-palmitoyl d_13_-lysoPC and d_13_-palmitoyl-glutaryl PC (PGPC), for use as probes during LC-MS/MS analysis. Our data showed that lysoPC was able to transfer spontaneously from LDL to HDL. In addition, part of the lysoPC was re-acylated by the action of lecithin-cholesterol acyltransferase (LCAT) to produce diacyl-PC. Moreover, short chain-oxPC was also capable of transferring between lipoproteins and was susceptible to hydrolysis by Lp-PLA_2_. These observations suggest that the oxPC and lysoPC species can be metabolized by interactions between oxLDL and HDL during the oxidation of LDL in vivo.

## 2. Materials and Methods

### 2.1. Materials

1,2-Dipalmitoyl-*sn*-glycero-3-phosphocholine-1,1,2,2-d_4_-*N,N,N*-trimethyl-d_9_ (16:0-16:0 d_13_-PC) and PC standards (1,2-didecanoyl-*sn*-glycero-3-phosphocholine (DDPC) and 1-pentadecanoyl-2-hydroxy-*sn*-glycero-3-phosphocholine (15:0-lysoPC)) were purchased from Avanti Polar Lipids, Inc. (Alabaster, AL, USA). Phospholipase A_2_ (PLA_2_) from bee venom was purchased from Sigma-Aldrich Co. (St. Louis, MO, USA). Acetonitrile, 2-propanol, ultrapure water (LC-MS grade), methanol, chloroform, ammonium formate and 5,5′-dithiobis (nitrobenzoic acid) (DTNB) were purchased from Fuji Film Wako Chemical Industries (Osaka, Japan). 4-(2-Aminoethyl)-benzenesulfonyl fluoride hydrochloride (pefabloc) was purchased from Roche Diagnostics (Basel, Switzerland).

### 2.2. Preparation of Human Lipoproteins

This study was conducted in accordance with the principles of the Declaration of Helsinki and approved by the Ethical Committee of Showa University (no. 214). Human lipoproteins were prepared from plasma by potassium bromide stepwise density gradient centrifugation [19]. Briefly, plasma was separated from human blood from healthy volunteers by centrifugation at 700× *g* for 15 min. EDTA was added to the resulting plasma (final concentration 0.25 mM). Chylomicron, VLDL, and IDL were then removed by centrifugation at 100,000 rpm (453,000× *g*) for 2.5 h in 250 µM EDTA/PBS. LDL was separated by an additional ultracentrifugation at 100,000 rpm for 2.5 h with addition of KBr to adjust the density to 1.063 g/mL. Then HDL was recovered by ultracentrifugation at 100,000 rpm for 4 h after adjusting the density to 1.21 g/mL. LDL and HDL were dialyzed against PBS containing 0.25 mM EDTA to remove KBr. The protein concentrations of the LDL and HDL fractions were determined by the bicinchoninic acid (BCA) method using BSA as a standard.

### 2.3. Preparation of 16:0 d_13_-lysoPC from 16:0-16:0 d_13_-PC

Dipalmitoyl d_13_-PC in chloroform (1.5 µmol) was dried under N_2_ gas and dispersed in 1200 µL of 0.1% ammonium formate at 60 °C by sonication. The PC was incubated with bee venom PLA_2_ in the presence of calcium chloride (final 4 µM) for 2 h at 37 °C. The resulting lipids, including 16:0 d_13_-lysoPC, were extracted using the Bligh and Dyer method [20]. The hydrolysis of 16:0-16:0 d_13_-PC and the release of 16:0 d_13_-lysoPC was confirmed by thin layer chromatography using a solvent system of chloroform/methanol/water 65:35:8 (*v*/*v*/*v*). The 16:0 d_13_-lysoPC was purified by high-performance liquid chromatography (HPLC) (Waters 1525) on a reversed-phase column (Capcell Pak C18, 4.6 × 250 mm) at 1 mL/min. Mobile phase A was comprised of acetonitrile/water 1:1 (*v*/*v*) containing 5 mM ammonium formate. Mobile phase B was comprised of acetonitrile/2-propanol 1:9 (*v*/*v*) containing 5 mM ammonium formate. The following gradient conditions were used: 30% B to 100% B over 60 min, 100% B over 5 min, and a return to 30% over 5 min, followed by 30% B over 5 min. The samples were dissolved in 30% B before being injected into the HPLC system. The elution profile was monitored by UV absorbance at 206 nm. The high-purity (>99%) of 16:0 d_13_-lysoPC was validated by MS.

### 2.4. Preparation of d_13_-PGPC

Using *N,N*-dimethyl-4-aminopyridine as a catalyst, d_13_-PGPC was synthesized by a condensation reaction of 16:0 d_13_-lysoPC (1 mg) and glutaric anhydride under anhydrous conditions according to a previous report [21], with some modifications. d_13_-PGPC was purified by HPLC under the same solvent system and then validated using mass spectrometry.

### 2.5. LDL Labeling with 16:0 d_13_-lysoPC

LDL (1 mg protein in 250 µM EDTA/PBS) was incubated with 16:0 d_13_-lysoPC (13 nmol in 20 μL ethanol) under argon gas in a shaded tube at 37 °C overnight. The 16:0 d_13_-lysoPC-labeled LDL was recovered by ultracentrifugation after adjusting the density (d = 1.063) with KBr followed by dialysis against PBS containing 0.25 mM EDTA, and its protein concentration was determined using the BCA method.

### 2.6. LDL Labeling with d_13_-PGPC

To suppress the degradation of d_13_-PGPC in LDL, the Lp-PLA_2_ enzyme activity in LDL was inhibited by pretreatment with pefabloc. LDL (1 mg protein) was treated with 0.5 mM pefabloc under argon gas in a shaded tube at 37 °C for 30 min. The inhibition of Lp-PLA_2_ activity by pefabloc was measured using a platelet-activating factor (PAF) analog with a 4-nitrophenyl group as the substrate [22]. This LDL solution was incubated with 1.3 nmol d_13_-PGPC under argon gas in a shaded tube at 37 °C for 30 min. The d_13_-PGPC-labeled LDL was recovered and its protein concentration was measured as described above.

### 2.7. Inhibition of Lp-PLA_2_ or LCAT Activity in HDL

Lp-PLA_2_ or LCAT activity in HDL was suppressed by pretreatment with each inhibitor [23,24]. HDL (1 mg protein) was treated with 2.5 mM pefabloc or 1 mM DTNB to inhibit Lp-PLA_2_ or LCAT activity, respectively, under argon gas in a shaded tube at 37 °C for 30 min. The pretreated HDL solutions were kept at 4 °C.

### 2.8. Assessment of lysoPC Transfer and PC Generation

An aliquot (0.1 mL) of 16:0 d_13_-lysoPC labeled LDL (100 µg) and HDL (100 µg) were co-incubated in 250 µM EDTA/PBS under argon gas in shaded tubes at 37 °C for up to 4 h. Following incubation, the lipoproteins were re-isolated by ultracentrifugation after adjusting to a final density of 1.063 g/mL using KBr solution (115,000 rpm, 4 °C, 1 h 15 min). The LDL was recovered in two fifths of the volume from the top of the tube. For HDL, the lower two-fifths of the tube was recovered [16]. The re-isolated HDL and re-isolated labeled LDL showed no cross-contamination of apoA1 and apoB by SDS-PAGE. On the other hand, labeled LDL and HDL and 10–20% of total cholesterol were recovered from the internal layer of the tube.

Lipids were extracted from re-isolated lipoproteins by the Bligh and Dyer method using chloroform and methanol containing 10 nM DDPC and 10 nM 15:0-lysoPC as internal standards, respectively. The samples were dried under N_2_ gas, dissolved in 100 µL chloroform/methanol 1:1 (*v*/*v*), and stored at −80 °C.

### 2.9. Assessment of LCAT Function

Palmitoyl d13-lysoPC-labeled LDL (100 µg protein) and HDL (100 µg protein) were co-incubated with 1 mM DTNB in 250 µM EDTA/PBS under argon gas at 37 °C in shaded tubes for 2 h. Following incubation, the lipoproteins were re-isolated and total lipids were extracted as described above.

### 2.10. Assessment of PGPC Metabolism

LDL labeled with d_13_-PGPC (100 µg protein) and inhibitor-treated HDL or non-treated HDL (0, 50, or 100 µg protein) were co-incubated in 250 µM EDTA/PBS under argon gas in shaded tubes at 37 °C for 30 min. Following incubation, the lipoproteins were re-isolated and the total lipids were extracted, as described above.

### 2.11. LC-MS/MS Analysis of Molecular Species d_13_-Labeled PCs

The molecular species of lipids were separated simultaneously, as described previously [17], using a Shimadzu Prominence HPLC system coupled to a QTRAP5500 triple quadruple mass spectrometer (AB SCIEX). Briefly, liquid chromatography separation was performed on a Mightysil RP-18MS column (2.0 × 150 mm; Kanto Kagaku, Tokyo, Japan) at 0.2 mL/min. Mobile phase A was comprised of acetonitrile/water 1:1 (*v*/*v*) containing 5 mM ammonium formate, while mobile phase B was comprised of acetonitrile/2-propanol 1:9 (*v*/*v*) containing 5 mM ammonium formate. The following gradient conditions were used: 30% B to 100% B over 60 min, 100% B over 10 min, and a return to 30% over 5 min, followed by 30% B over 5 min. The mass spectrometer was operated in positive ion mode with an ion-spray voltage of 5.5 kV. The collision energy was set at 8.0 V for d_13_-labeled PC species.

For each analysis, the samples were dried under N_2_ gas, dissolved in 100 µL of mobile phase solvent A/B = 7:3 (*v*/*v*) and 10 µL of the sample was injected. Deuterium-labeled PC species were detected by multiple reaction monitoring (MRM), in which 22 selected ions released a fragment ion of deuterium phosphorylcholine (*m*/*z* 197.1) (Table 1). The relative amounts of PC species were calculated semi-quantitatively using DDPC and 15:0-lysoPC as an internal standard (2 pmol each).

### 2.12. Statistical Analysis

Statistical analysis was performed using the JMP Pro13 software. Comparison with the control was analyzed using Dunnett’s test. Statistical differences within sample groups were determined using paired *t*-test. A value of *p* < 0.05 was considered statistically significant.

## 3. Results

### 3.1. Transfer and Metabolism of d_13_-lysoPC

**Figure 1:**
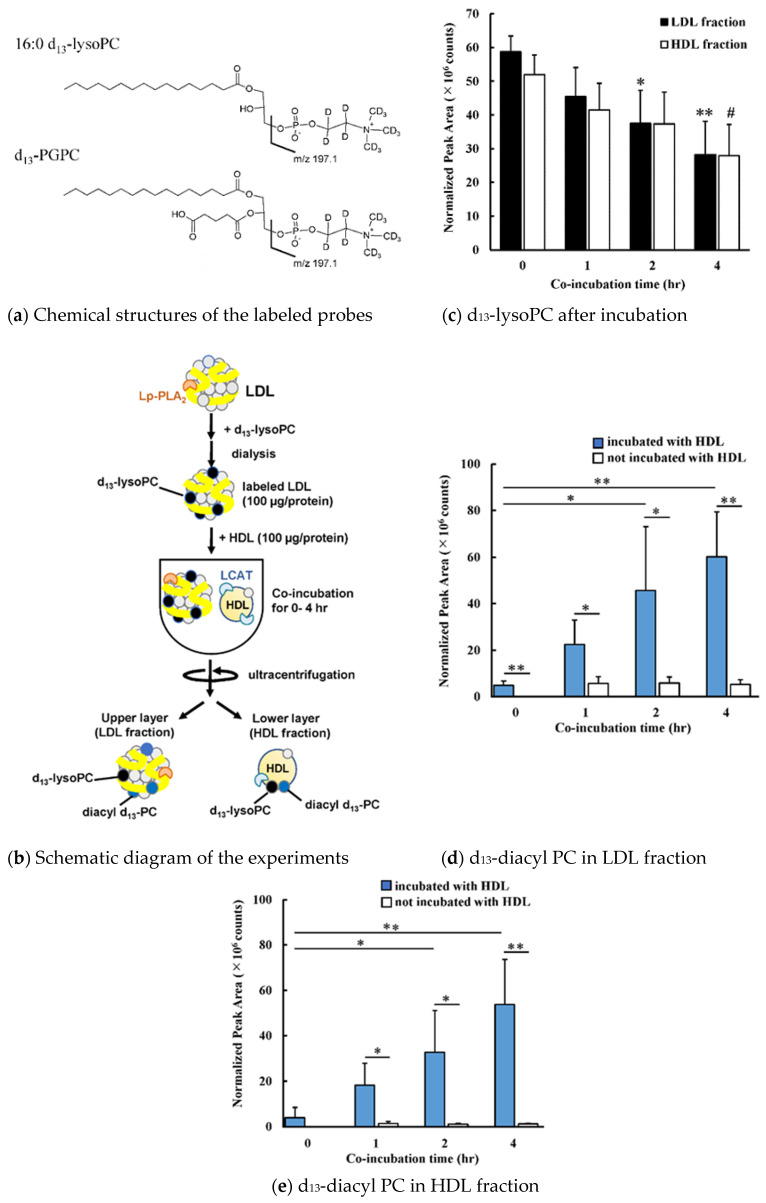
(**a**) Chemical structure of 16:0 d_13_-lysoPC (*m*/*z* 509.3) and d_13_-PGPC (*m*/*z* 623.6), which generated a d_13_-phosphorylcholine-specific fragment ion of *m*/*z* 197.1. (**b**) Schematic diagram of the experimental procedure of lysoPC transfer from LDL to HDL. (**c**) Time-course change of d_13_-lysoPC content in LDL (solid bars) and HDL (open bars) fractions when d_13_-lysoPC labeled-LDL was incubated with HDL for up to 4 h. Bar graphs show the peak area (cps) of 1-palmitoyl-d_13_-lysoPC. * *p* < 0.05, ** *p* < 0.01 vs. LDL, # *p* < 0.05 vs. HDL fractions at an incubation time of 0 h. (**d**,**e**) Time-dependent production of diacyl d_13_-PC species in the LDL (**d**) and HDL (**e**) fractions. The d_13_-lysoPC labeled-LDL was incubated with HDL (solid bars) or without HDL (open bars) for up to 4 h. The bar graph shows the total amount of 15 species of diacyl-d_13_-PC (cps) detected by LC-MS/MS. * *p* < 0.05, ** *p* < 0.01. Results represent the mean (SD) of four independent experiments.

LDL reconstituted with a stable isotope-labeled probe was prepared by adding d_13_-lysoPC to LDL and incubating overnight, followed by ultracentrifugation. Reconstituted LDL was incubated with or without HDL for up to 4 h, then LDL and HDL were separated by ultracentrifugation (Figure 1a,b). Since the sample volume was small (0.5 mL) a part of HDL or LDL was distributed in the intermediate fraction, however, recovery of apoB in the bottom fraction or apoA1 in the top fraction was minimal (Supplementary Figure S1). The profile of the stable isotope-labeled PC species in the LDL fraction was quantitated by LC-MS/MS. The amount of d_13_-lysoPC in the LDL fraction was found to decrease in parallel with the incubation time (Figure 1c). Under the experimental conditions, almost the same amounts of d_13_-lysoPC were recovered in both LDL and HDL, suggesting that lysoPC can be rapidly transferred from LDL to HDL. It is worth noting that, although samples were handled as quickly as possible, some time was spent handling the samples before starting the centrifugation of the HDL and d_13_-labeled LDL mixtures. Therefore, even in the “0 h incubation” samples, there might have been sufficient time for d_13_-lysoPC to have moved between lipoproteins. After incubating LDL with HDL for 4 h, about 50% of d_13_-lysoPC was recovered in LDL. This decrease in d_13_-lysoPC was observed only when HDL was added to LDL (Supplementary Figure S2).

During the incubation of HDL and d_13_-lysoPC-containing LDL, d_13_-labeled diacyl-PC species were generated in a time-dependent manner. This diacyl-PC formation was dependent on the presence of HDL (Figure 1d). Interestingly, diacyl-PC was found both in the upper layer (LDL) and lower layer (HDL).

**Figure 2:**
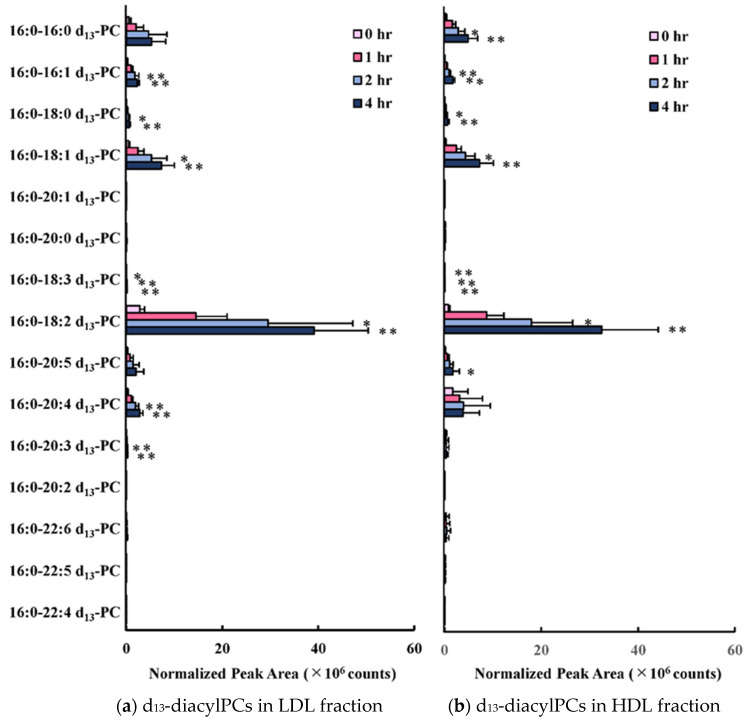

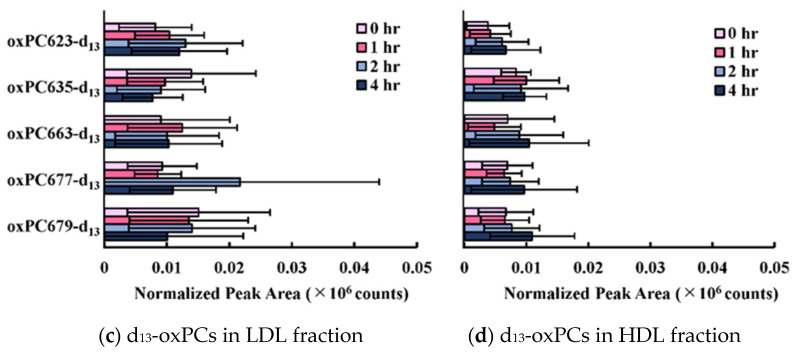
The time course changes in the amount of 15 diacyl d_13_-PC species (**a**,**b**) and 5 d_13_-oxPC species (**c**,**d**) in the LDL and HDL fractions, respectively. The d_13_-lysoPC labeled-LDL was incubated with HDL for up to 4 h. Bar graphs show the relative peak area (cps) of PC and oxPC species derived from 1-palmitoyl-d_13_-lysoPC. Results represent the mean (SD) of four independent experiments. * *p* < 0.05, ** *p* < 0.01 vs. co-incubation time at 0 h.

Figure 2 shows the profiles of molecular species containing the d_13_-stable isotope-labeled group. The main diacyl-PC species was 16:0-18:2-d_13_-PC (*m*/*z* 771.6), although several other species were also detected. Only a small difference was observed between the PC profiles derived from 1-palmitoyl-d_13_-lysoPC in LDL and HDL. The oxidation of the diacylPC and the transfer of oxidized acyl moiety to 1-palmitoyl- d_13_-lysoPC in LDL and HDL were minimal, since the peak areas of oxPC derivatives were two orders smaller than those of the diacyl PC species formed.

**Figure 3:**
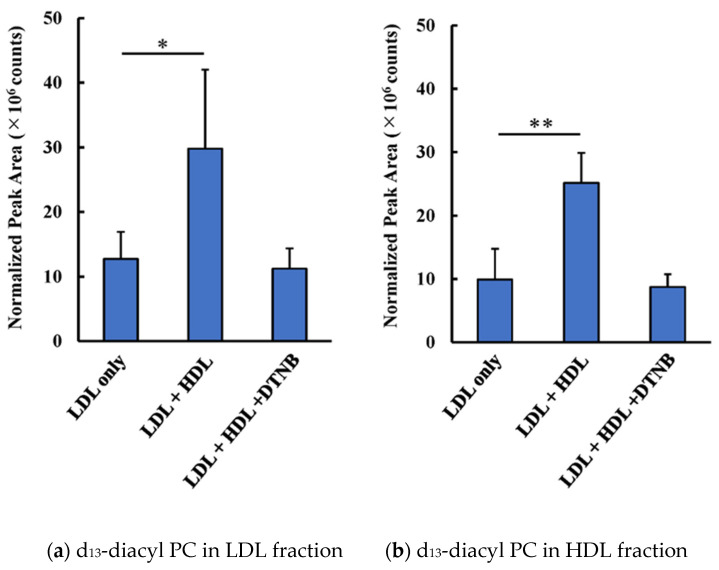
Effect of inhibitors for LCAT on d_13_-lysoPC metabolism. LDL preloaded with d_13_-lysoPC was incubated with HDL pretreated either with or without DTNB. Bar graphs illustrate changes in the total amount of 15 diacyl d_13_-PC species in the LDL (**a**) and HDL (**b**) fractions after ultracentrifugation. Results represent the mean (SD) of five independent experiments. * *p* < 0.05, ** *p* < 0.01 vs. LDL only.

Diacyl-PC formation was completely abolished by the addition of DNTB, which binds with the sulfhydryl group to inhibit LCAT (Figure 3). When d_13_-lysoPC-labeled LDL was incubated with or without HDL for 2 h, the amount of d_13_-diacyl-PC in either LDL or HDL increased 2- to 3-fold in the presence of HDL. However, diacyl-PC formation was not observed when HDL was pretreated with DTNB.

### 3.2. Transfer and Metabolism of d_13_-PGPC

**Figure 4:**
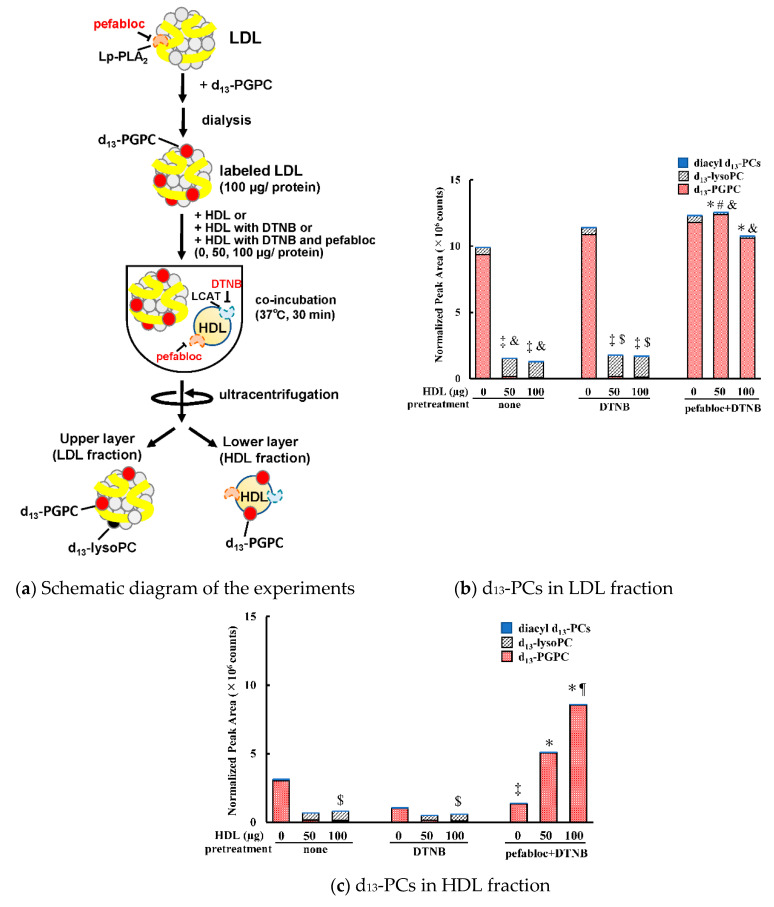
(**a**) Schematic diagram of the experimental procedure of d_13_-PGPC transfer from reconstituted oxLDL to HDL. LDL preloaded with d_13_-PGPC in the presence of pefabloc was further incubated for 30 min at 37 °C with HDL pretreated either with pefabloc or with pefabloc and DTNB or without inhibitors. (**b**,**c**) Stacked bar graph showing the peak areas (cps) for d_13_-PGPC, 16:0 d_13_-lysoPC, and the total amounts of 15 diacyl d_13_-PC species in the re-isolated LDL fraction (**b**) and re-isolated HDL fraction or lower layer (**c**). Data represent the mean of three independent experiments. Symbols: * *p*, #, or ^¶^ indicates *p* < 0.05 between values for d_13_-PGPC, 16:0 d_13_-lysoPC, or diacyl d_13_-PCs compared with those in not pretreated with inhibitors (none), respectively. ^‡^, ^&^, or ^$^ indicates *p* < 0.05 between values for d_13_-PGPC, 16:0 d_13_-lysoPC, or diacyl d_13_-PCs compared with those in the samples not incubated with HDL (HDL 0 µg), respectively.

We reconstituted LDL containing a synthetic short-chain oxPC, d_13_-PGPC, as a model of oxLDL to evaluate the movement and metabolism of oxPC on lipoproteins. The organic synthesis of d_13_-PGPC was carried out via a condensation reaction between d_13_-lysoPC and glutaric anhydride. It is worth noting that the pefabloc was necessary during reconstitution, since PGPC was quickly destroyed during pretreatment without this inhibitor. HDL was pretreated with either DTNB or DTNB plus pefabloc, or no pretreatment. The reconstituted oxLDL was then incubated with either of these three HDL preparations for 30 min. Then, the LDL and HDL fractions were separated by ultracentrifugation. The molecular species of the d_13_-labeled PC derivatives were quantitated by LC-MS/MS (Figure 4). The addition of non-treated HDL to reconstituted oxLDL reduced d_13_-PGPC almost completely, and only a small amount of lysoPC was detected. Approximately 10–20% of PGPC and lysoPC was detected in the lower layer (HDL). However, only a small amount of d_13_-labeled-diacyl-PC was generated in LDL and HDL.

When HDL pretreated with both pefabloc and DTNB was added to d_13_-PGPC labeled reconstituted oxLDL, the production of lysoPC was almost abolished and the distribution of d_13_-PGPC to HDL was increased, depending on the amount of HDL added. These results suggest that the Lp-PLA_2_ present in both HDL and LDL contributes to the hydrolysis of oxPC molecules. The data also showed that PGPC can be transferred between LDL and HDL.

## 4. Discussion

In this study, we evaluated the behavior of lysoPC and PGPC, which are formed in oxLDL, in the presence of LDL and HDL by using stable isotope-labeled molecules. PGPC is readily hydrolyzed by Lp-PLA_2_ and able to transfer to HDL from LDL. Likewise, lysoPC can be quickly transferred to other lipoproteins and metabolized by LCAT to form diacyl-PC. LDL and HDL are the major lipoproteins in human circulation; therefore, interactions between LDL and HDL particles are likely to occur during oxLDL formation under in vivo conditions.

**Figure 5:**
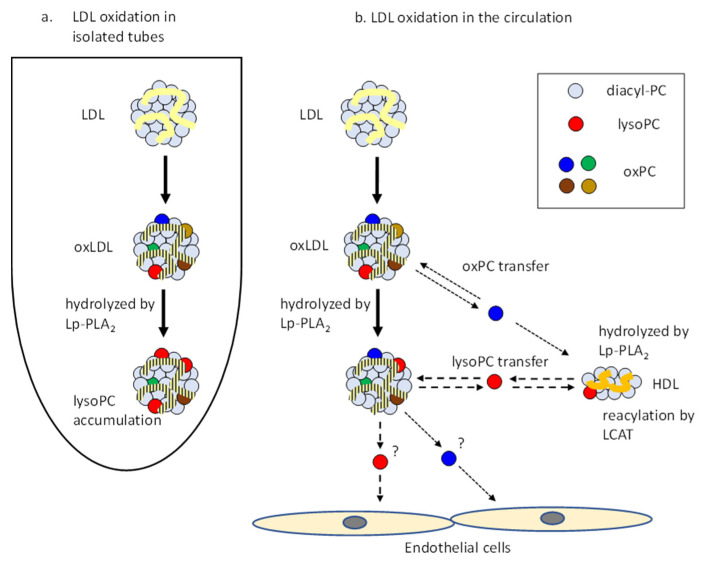
Schematic illustration of the transfer and metabolism of phospholipids in lipoproteins upon oxidative modification. (**a**) Oxidized materials and lysoPC produced by Lp-PLA_2_ accumulated in the LDL particle when oxidized under closed conditions. (**b**) HDL acts as a reservoir of lysoPC and oxPCs, as well as providing enzymes for their metabolism. In particular, LCAT contributes to the reproduction of diacyl PC from lysoPC. LysoPC and oxPCs are not only transferred between lipoproteins but also to cellular membranes in the vessel wall tissues.

Copper-induced oxLDL is widely used as an oxLDL model. Various oxPC species and lysoPC are generated in oxLDL. A previous study found that a large amount of lysoPC (as much as 30% of all PC molecules) accumulated in oxLDL after 3 h of incubation of LDL with copper sulfate [17]. However, we recently isolated and characterized in vivo oxLDL from human plasma and found that lysoPC did not increase in oxLDL in vivo [10]. The present study provides an explanation as to why lysoPC does not accumulate in oxLDL in the circulation. LysoPC and short-chain oxPC can be transferred to other lipoproteins, including HDL, and are enzymatically metabolized in the presence of HDL. When LDL particles were oxidatively modified in circulation or in atherosclerotic plaques, oxPC and lysoPC molecules were easily transferred to other lipoproteins. In addition, they were enzymatically metabolized, and did not accumulate in LDL. On the other hand, when isolated LDL was incubated with copper ion, Lp-PLA_2_ readily hydrolyzed oxPC, and the resulting lysoPC was transferred between LDL particles, but was not further metabolized (Figure 5).

HDL is atheroprotective since it transfers excess cholesterol from peripheral tissues back to the liver, as well as having antioxidative properties. ApoA1, the major protein constituent of HDL, has several reactive amino acid residues that reduce oxidized molecules; however, apoA1 itself is modified during oxidation [24,25]. Hazen and colleagues reported that oxHDL was present in both the circulation and atherosclerotic plaques [9,26]. HDL was found to be highly susceptible to oxidation, particularly to modification with acrolein [27]. Our study demonstrated that the in vivo oxLDL isolated from human plasma contained oxHDL particles in the same fraction [10]. Based on these findings, it is reasonable to assume that the interaction between LDL and HDL occurs during the oxidative modification of LDL, and both LDL and HDL contribute to the formation of atherosclerotic lesions. Thus, the present study supports the hypothesis that the transfer and metabolism of PC derivatives occurs in the presence of HDL.

Lp-PLA_2_ hydrolyzes oxPC to form lysoPC in oxLDL [16,18]. In this study, this enzyme was found to be very efficient, since all d_13_-labeled-PGPC was completely destroyed during preincubation in the absence of pefabloc. Thus, control experiments without the inhibitor cannot be performed. In addition, our results showed that LCAT contributed to the re-acylation of lysoPC. It was shown that the LCAT in HDL is also involved in the metabolism of PGPC; however, the final products of d_13_-PGPC seem to be diverse. PGPC may be converted to hydrophilic derivatives by the phospholipase A_1_ activity of LCAT [28]. Alternatively, oxPC, including PGPC, may suppress lysoPC-acyltransferase activity I, namely the esterification of lysoPC with a long chain acyl group, of LCAT [29].

LCAT is known to generate cholesterol ester and catalyze the transacylation reaction between various substrates, such as PC and lysoPC [23,30,31]. If the reacylation of lysoPC is the reverse reaction of cholesterol esterification, free cholesterol is generated in HDL concomitant with diacyl PC. Based on the lipid compositions of LDL and HDL in the literature, the ratio of free cholesterol to total cholesterol can be reduced from 25% to 36%. Such reduction in HDL may improve the efflux of cholesterol from cells. LCAT may be anti-atherogenic by two-fold, namely, by eliminating lysoPC molecules by reacylation and by maintaining the efflux of cholesterol from atherosclerotic lesions.

The spontaneous transfer of lysoPC between membranes was characterized in a previous study using liposomes, in which the transfer of lysoPC between lipoproteins and liposomes was also demonstrated [32]. In the present study, we used a stable isotope-labeling technique and MS to demonstrate not only the quick transfer of lysoPC between LDL and HDL but also that the enzymatic metabolism of these phospholipids occurs simultaneously in lipoproteins. Ramiena, et al. previously demonstrated the transfer of long-chain oxPC and oxidized cholesterol from oxLDL to HDL [24], but did not analyze lysoPC and the contribution of enzymatic reactions. In our experimental conditions, no plasma proteins were added to the assay mixture; however, the potential contribution of any lipoprotein-associated lipid-interacting proteins, such as CETP, cannot be denied. Transferable oxPC in lipoproteins can be recognized by the scavenger receptor CD36 [33]. The transfer of lipids between lipoproteins and between lipoproteins and various cells may be common activities under physiological conditions.

A stable isotope-labeled PGPC was introduced as one of the short-chain oxPC products, since the synthesis of this compound is a one-step reaction, which is slightly simpler than other oxPC species. We assumed that the short-chain oxPC species could behave similarly to some extent; however, the detailed characteristics of each oxPC species should be examined in a separate study. It would also be of interest to determine whether oxPC and lysoPC can be transferred from oxLDL to the membranes of vascular cells, in addition to lipoproteins in vivo.

In conclusion, during the oxidative modification of LDL, the interaction between HDL and LDL facilitates the transfer and metabolism of short-chain oxPC and lysoPC molecules. This allows for the oxPC and lysoPC content of oxLDL particles formed in vivo to be maintained at a low level.

## Figures and Tables

**Table 1:** List of 20 PC molecular species expected to be generated from 1-palmitoyl d_13_-lysoPC monitored by LC-MS/MS.

*m*/*z*	Molecular Species	*m*/*z*	Molecular Species
S/MUFA-d_13_-PC		Short chain d_13_-oxPC	
747.6	16:0-16:0 d_13_-PC	623.6	1-palmitoyl-2-glutaroyl d_13_-PC
745.6	16:0-16:1 d_13_-PC	635.6	1-palmitoyl-2-(7-oxo-heptanoyl) d_13_-PC
775.6	16:0-18:0 d_13_-PC	663.6	1-palmitoyl-2-(9-oxo-nonanoyl) d_13_-PC
773.6	16:0-18:1 d_13_-PC	677.6	1-palmitoyl-2-(5-oxo-octenoyl) d_13_-PC
801.6	16:0-20:1 d_13_-PC	679.6	1-palmitoyl-2-azelaoyl d_13_-PC
803.6	16:0-20:0 d_13_-PC		
		d_13_-LysoPC	
PUFA-d_13_-PC		509.3	1-palmitoyl d_13_-lysoPC
769.6	16:0-18:3 d_13_-PC		
771.6	16:0-18:2 d_13_-PC		
793.6	16:0-20:5 d_13_-PC		
795.6	16:0-20:4 d_13_-PC		
797.6	16:0-20:3 d_13_-PC		
799.6	16:0-20:2 d_13_-PC		
819.6	16:0-22:6 d_13_-PC		
821.6	16:0-22:5 d_13_-PC		
823.6	16:0-22:4 d_13_-PC		

In MRM mode, corresponding to the *m*/*z* values with a fragment ion of 197 were selectively detected.

## Supplementary Materials

The following are available online at https://www.mdpi.com/2076-3921/9/11/1045/s1, Figure S1: Selective recovery of LDL and HDL by ultracentrifugation. After incubating LDL (100 mg) and HDL (100 mg) for 2 h (total volume 0.5 mL), they were separated by ultracentrifugation after adjusting d = 1.063 using KBr. Figure S2: Addition of HDL decreased d13-lysoPC labeled in LDL. Recovery of d13-lysoPC was expressed as relative ratio to the total d13-labeled PC (21 species) in the re-isolated LDL fraction.
