# Supplementary material for: Transfer and Enzyme-Mediated Metabolism of Oxidized Phosphatidylcholine and Lysophosphatidylcholine between Low- and High-Density Lipoproteins
Source: Antioxidants (Basel). 2020 Oct 26;9(11):1045. doi: 10.3390/antiox9111045 (PMC7712993; doi:10.3390/antiox9111045)
Supplement: Supplementary file 1 [file antioxidants-09-01045-s001.zip › Suppl. Fig. S1-Edited.pptx]

## Slide 1
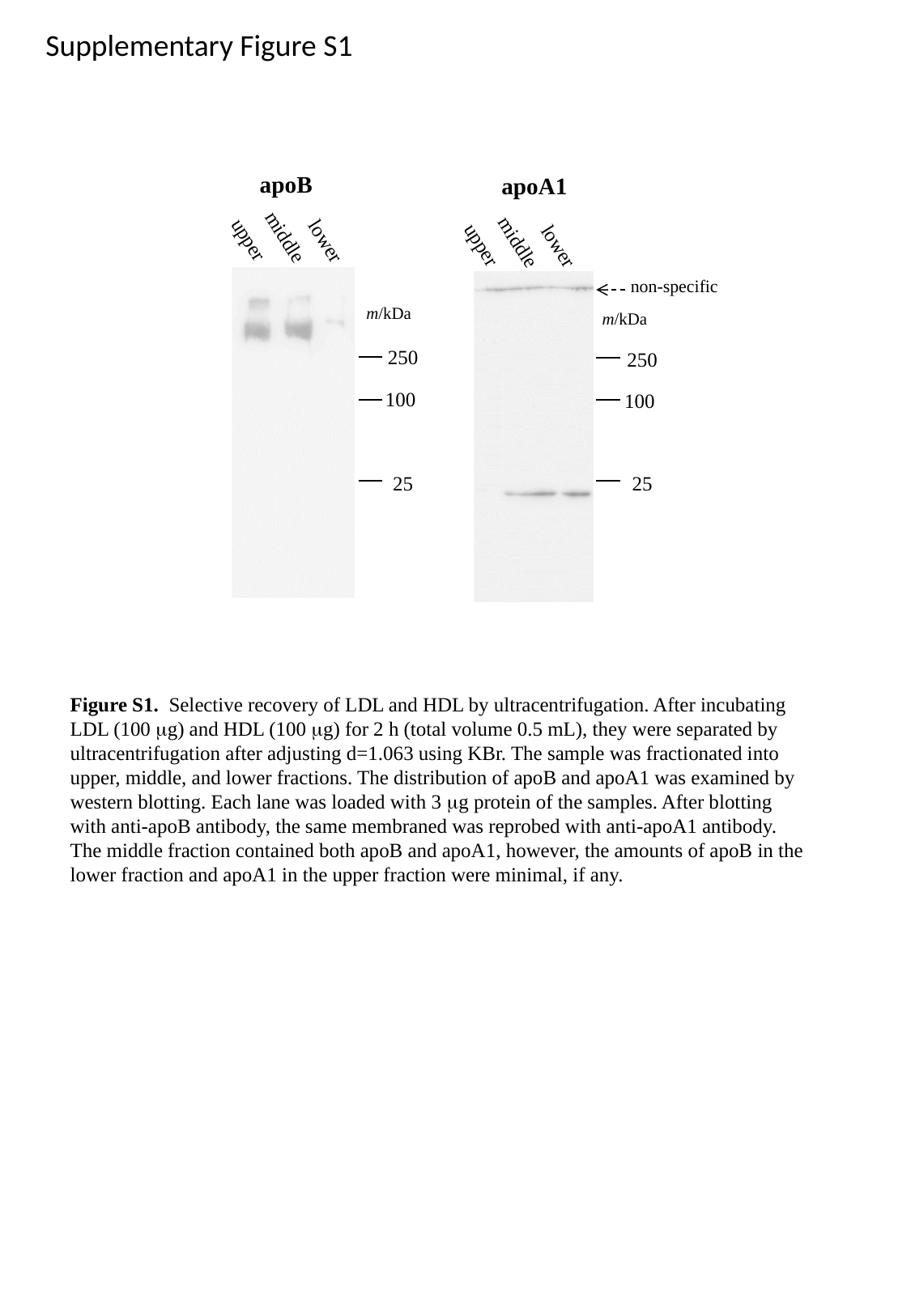

Supplementary Figure S1
apoB
apoA1
middle
upper
lower
middle
upper
lower
non-specific
m/kDa
m/kDa
250
250
100
100
25
25
Figure S1. Selective recovery of LDL and HDL by ultracentrifugation. After incubating LDL (100 mg) and HDL (100 mg) for 2 h (total volume 0.5 mL), they were separated by ultracentrifugation after adjusting d=1.063 using KBr. The sample was fractionated into upper, middle, and lower fractions. The distribution of apoB and apoA1 was examined by western blotting. Each lane was loaded with 3 mg protein of the samples. After blotting with anti-apoB antibody, the same membraned was reprobed with anti-apoA1 antibody. The middle fraction contained both apoB and apoA1, however, the amounts of apoB in the lower fraction and apoA1 in the upper fraction were minimal, if any.
