# Supplementary material for: Transfer and Enzyme-Mediated Metabolism of Oxidized Phosphatidylcholine and Lysophosphatidylcholine between Low- and High-Density Lipoproteins
Source: Antioxidants (Basel). 2020 Oct 26;9(11):1045. doi: 10.3390/antiox9111045 (PMC7712993; doi:10.3390/antiox9111045)
Supplement: Supplementary file 1 [file antioxidants-09-01045-s001.zip › Suppl. Fig. S2-Edited.pptx]

## Slide 1
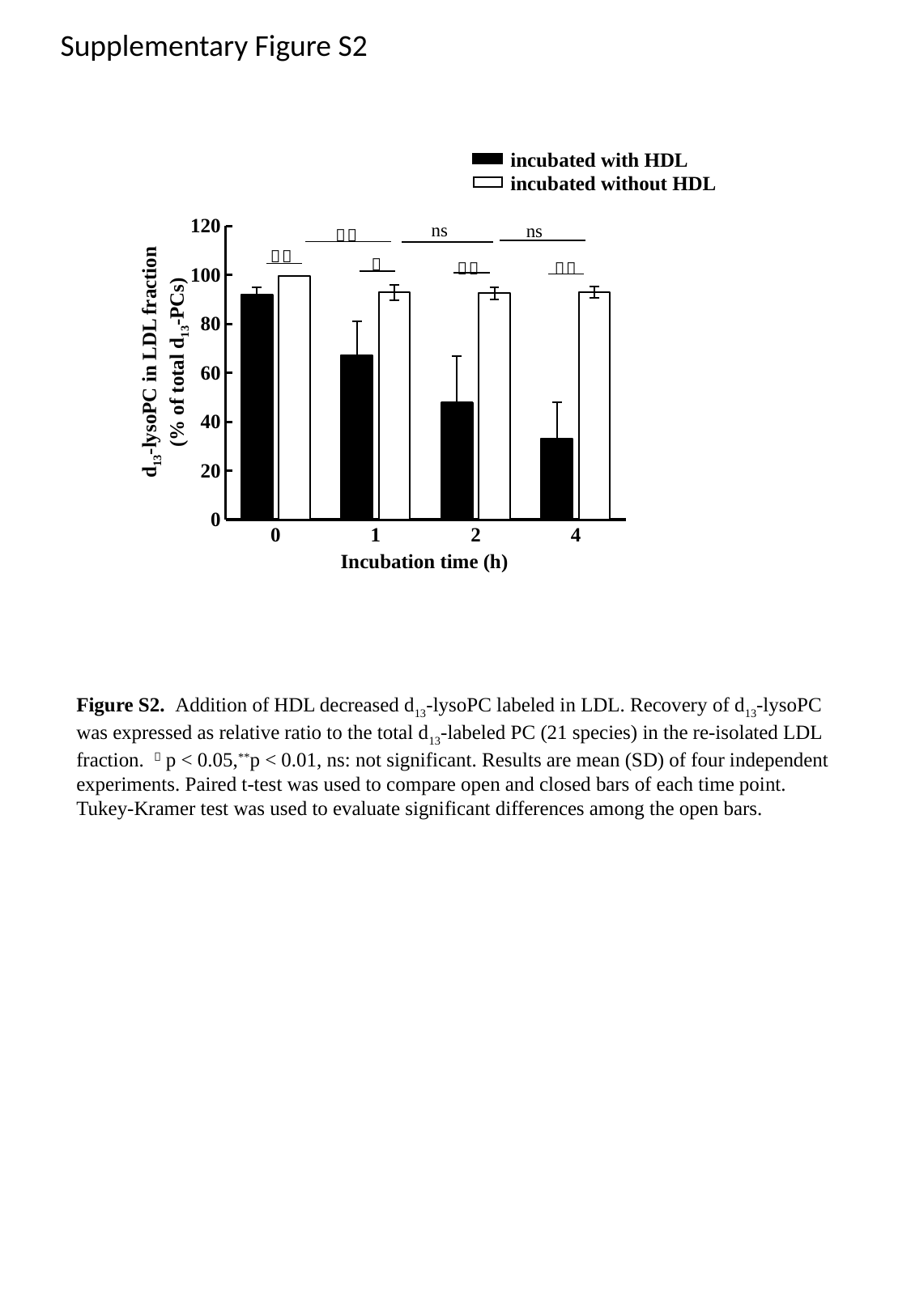

Supplementary Figure S2
incubated with HDL
incubated without HDL
### Chart
| Category | HDL(+) | HDL(-) |
|---|---|---|
| 0 | 92.11540103977035 | 99.5728537950755 |
| 1 | 67.08838171721457 | 92.92976504315985 |
| 2 | 47.81548671706348 | 92.51640740268704 |
| 4 | 32.98332172875011 | 92.91867004654881 |ns
ns
＊
＊
＊
＊
＊
＊
＊
＊
＊
d13-lysoPC in LDL fraction
(% of total d13-PCs)
Incubation time (h)
Figure S2. Addition of HDL decreased d13-lysoPC labeled in LDL. Recovery of d13-lysoPC was expressed as relative ratio to the total d13-labeled PC (21 species) in the re-isolated LDL fraction. ＊p < 0.05,**p < 0.01, ns: not significant. Results are mean (SD) of four independent experiments. Paired t-test was used to compare open and closed bars of each time point. Tukey-Kramer test was used to evaluate significant differences among the open bars.
